# Mechanical stress contributes to ligamentum flavum hypertrophy by inducing local inflammation and myofibroblast transition in the innovative surgical rabbit model

**DOI:** 10.3389/fimmu.2025.1541577

**Published:** 2025-04-15

**Authors:** Qinghong Ma, Xincheng Feng, Yongxin Chen, Jue Zhang, Chao Sun

**Affiliations:** Department of Spine Surgery, The Affiliated Jiangning Hospital of Nanjing Medical University, Nanjing, Jiangsu, China

**Keywords:** TGF-β1, ligamentum flavum, fibrosis, mechanical stress, rabbit model

## Abstract

**Background:**

Lumbar spinal canal stenosis (LSCS) ranks as a prevalent spinal disorder in senior populations. Ligamentum flavum hypertrophy (LFH) is a significant feature of LSCS, yet its cause is unclear. The purpose of this study was to create a novel animal model for LFH and explore the pathological mechanisms involved.

**Methods:**

A novel rabbit model for intervertebral mechanical stress concentration was established through posterolateral fusion using steel wire. Radiological analysis and biological validation were used to determine the crucial role of mechanical stress in LFH and explore the effect of this animal model.

**Results:**

After 12 weeks, the LF subjected to mechanical stress concentration exhibited a disruption and reduction in elastic fibers, collagen accumulation, increased thickness of LF, elevated LF cells, and increased levels of certain factors related to fibrosis and inflammation. These findings were histologically consistent to those found in human LFH. Furthermore, *in vitro*, mechanical stretch was discovered to enhance the conversion of fibroblasts into myofibroblasts by boosting TGF-β1 secretion in LF fibroblasts. In addition, compared to conventional internal fixation, this new surgical model provided advantages such as minor damage, decreased bleeding, and reduced technical difficulty and molding costs.

**Conclusion:**

This novel rabbit model is able to replicate the moderate pathological features of human LFH. Mechanical stress is an independent factor leading to LFH, which can promote the TGF-β1 secretion in LF cells and some inflammatory cells, subsequently induce the myofibroblast transition, and finally result in collagen accumulation and LF fibrosis.

## Introduction

As the population ages, lumbar spinal canal stenosis (LSCS) is emerging as the most prevalent spinal disorder among older adults (1). It is clear that the thickening of the ligamentum flavum (LF) plays a significant role in the onset of LSCS (2). Anatomically, the LF shields the posterior and lateral aspects of the dura sacs. Hypertrophy of the LF leads to spinal stenosis, and nerve root and cauda equina compression, resulting in spinal problems (3). Previously, despite a variety of studies examining LF degeneration, the molecular mechanisms remain unclear.

As of today, surgery is the main method of treating LSCS due to LFH (4). Controlling or delaying LFH is possible if its mechanism is thoroughly understood. This is an ideal treatment method with great economic and social benefits. A number of factors may contribute to the physiological and pathological mechanisms of LF hypertrophy, including age, physical activity, genetics, and mechanical stress (2). Most importantly, mechanical stress is thought to be one of the major causes (5, 6). In brief, it is recognized that segmental instability is an independent risk factor for LF thickening (7, 8). Histologically, mechanical stress accelerates the elastic fibers degeneration and causes the collagen accumulation (5, 9, 10). Furthermore, collagen synthesis was found to increase due to mechanical stretching force in the *in vitro* study (5, 9).

As such, a good understanding of the impact of mechanical stress on the LFH will help to enlighten its etiology. Previously, research conducted *in vivo* directly proves that mechanical stress can lead to LF hypertrophy (11, 12). However, the animal models were proved to have disadvantages of deep exposure, more bleeding, and high technical requirements for operator, which to some extent limits its application. Therefore, the construction of ideally animal models for lumbar LFH is still in the exploratory stage, and there is currently no recognized modeling method. Thus, it is necessary to develop a standard animal model to explore the role of mechanical stress on LFH and elucidate its molecular mechanisms. In the present study, we developed a rabbit model for LFH by employing an innovative internal fixation method and analyzed the influence of mechanical stress concentration on it. Moreover, we hoped to investigate the possible pathomechanism of LFH.

## Materials and methods

### Ethical approval

The Nanjing Medical University’s Ethics Committee approved this protocol. At 12 weeks after surgery, all rabbits were sacrificed, and the LF was harvested for further study. Samples of human LF were collected from the Orthopedics Department at the Affiliated Jiangning Hospital of Nanjing Medical University. Briefly, human LF was sourced from LSCS patients aged 55 to 65 years with an MRI-measured LF thickness exceeding 4mm, who underwent lumbar posterior decompressive laminectomy. Samples of non-hypertrophic LF were collected from patients with lumbar disc herniation (LDH) who were matched by age and sex, and demonstrated by MRI with LF thickness less than 4mm. All LF samples in this study were gathered from the L4/5 level (**Table 1**). Each patient gave written consent to participate in the study before being recruited. This work has been reported in line with the ARRIVE guideline (13).

**Table 1:** Comparison of data between two groups.

Index	LSCS group	LDH group	P value
Number of patients	10	10	
Mean age (years)	60.3 ± 3.5	57.5 ± 2.4	<0.05
Gender (male/female)	6/4	5/5	–
Level	L4/5	L4/5	–
LF thickness	4.95 ± 0.4	2.88 ± 0.2	<0.05
Fibrosis score	3.39 ± 0.22	1.35 ± 0.20	<0.05

### Animals and groups

**Figure 1:**
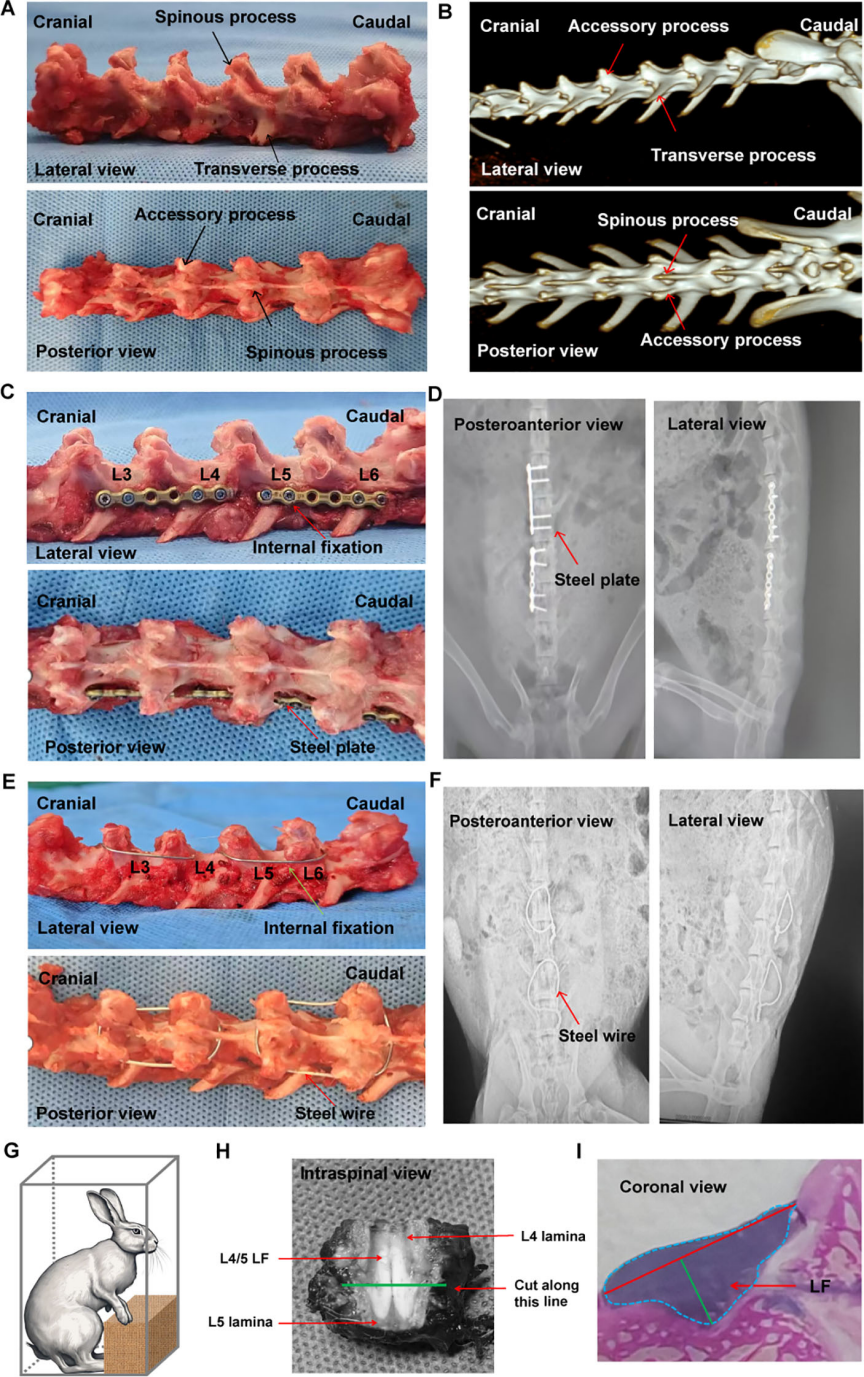
Examination of rabbit spinal structure, implant and internal fixation method, and LF thickness evaluation. **(A)** Anatomical features of the rabbit spine. **(B)** Three-dimensional spinal image taken before surgery. **(C)** Fused spine at L3/4 and L5/6 with a locking plate, viewed from the lateral and posterior side. **(D)** Standard X-ray images of the spine fused with internal fixation using a locking plate. **(E)** Fused spine at L3/4 and L5/6 reinforced with steel wire, visible from lateral and posterior perspectives. **(F)** Typical plain X-rays showing the fused spine with steel wire internal fixation. **(G)** Graphic depiction of the bipedal standing rabbit. **(H)** The cutting level was established by slicing a cross section at the midpoint between the upper and lower edges of LF. **(I)** Method for assessing the LF thickness. LF, ligamentum flavum.

Four groups were formed by randomly assigning 48 male New Zealand Rabbits aged 16 weeks, each weighing between 2.5 kg and 3.0 kg. The first group is the sham group with no internal fixation (Sham group n=12). The second group underwent the bipedal standing posture (BS group; n=12). In brief, the rabbits were positioned in a rectangular chamber to keep a bipedal standing posture and maintained in this position for 8 hours each day. The rectangular chambers measure 25cm in length, 20cm in width, and 45cm in height. Food and water were not provided while standing. The third group performed L3-4 and L5-6 posterolateral fusion with steel plate and removed the interspinous ligament to achieve mechanical stress concentration at L4-5 level (Locking Plate group; n=12) (**Figures 1C, D**). The forth group performed L3-4 and L5-6 posterior fusion with steel wire fixing spinous and accessory processes and also resected the interspinous ligament to form mechanical stress concentration in L4-5 level (Steel Wire group; n=12) (**Figures 1E, F**). The rabbits were kept individually in temperature- and humidity- controlled environments.

### Surgical procedure

Rabbits from Locking Plate group were prone-positioned after anesthesia with ketamine hydrochloride (30mg/kg) and xylazine (10mg/kg). As described in our previous study **^5-6^**, we performed L3-4 and L5-6 posterolateral fusion with steel plate and removed the interspinous ligament to obtain mechanical stress concentration in L4-5 level.

Rabbits in Steel Wire group were performed L3-4 and L5-6 posterior fusion with steel wire fixing spinous and accessory processes, and the interspinous ligament was also removed to focus mechanical stress at the L4-5 level. In brief, a 3 cm incision was made along the posterior median line under general anesthesia to reveal the spinous and accessory processes in the region of L3-L6. Subsequently, the electric drill penetrated at the root of the spinous process, and the needle wire was threaded out from the drilling site. Finally, it was tightened and fixed around the spinous process and mammillary process of the L3-4. Similarly, the procedure for fixing the L5-6 region was identical to that used for L3-4. (**Figures 1E, F**). Furthermore, additional L4-5 interspinal ligament resection was undergone after internal fixation. Finally, the incisions were sutured layer by layer with nylon after ensuring proper hemostasis and rinsing with saline solution.

### Radiological analysis

All the rabbits were taken anterioposterior and lateral X-ray at the time-point of pre-operation, post-operation, and 12 weeks after surgery. Over extension and over flexion X-ray of the lateral view of rabbit spine was conducted to assess the intervertebral motion range of L3-4, L4-5, and L5-6 under general anesthesia by utilizing a pressure measuring device (Nanjing Pharmaceutical Co., Ltd, Nanjing, China). The data were sent to a PACS software for analysis. Under anesthesia, the intervertebral motion range from L3-4 to L5-6 was examined through a lateral view of a manipulated dynamic X-ray with a pressure measuring device (Nanjing Medical University, Nanjing, China). The disk height at the anterior edge and the intervertebral range of motion were measured three times in the PACS system for all rabbits by a skilled spine surgeon, and the mean value was regarded as the ultimate result.

### LF sample collection

Samples of human LF were taken from the dorsal region at the L4/5 level. At 12 weeks after surgery, all the rabbits were euthanized, and LF samples from L4/5 were collected for further experiments. In this study, each sample was divided into three parts. One section of the sample was swiftly frozen and stored at -80°C for molecular biological analysis. The other part was fixed and then embedded for histopathological analysis. The leftover portion was promptly utilized for *in vitro* LF cell culture.

### LF cell isolation and culture

As soon as the samples of LF had been collected, they underwent washing with PBS (Gibco), were sliced into smaller pieces, and then digested with 0.2% collagenase for a duration of one hour. Subsequently, the pieces were cocultured with DMEM (Gibco) by adding fetal bovine serum and penicillin/streptomycin (Sigma). Proteintech antibodies against COL1A1 and COL3A1 were utilized for immunofluorescence staining to identify LF cells. Cells from the third passage were used in later experiments, requiring about 3 weeks to progress from primary to third -generation.

### LF cells subjected to mechanical stretching

In a specialized chamber from Beyotime, Beijing, China, LF cells were seeded at a concentration of 1×10^6^ cells per chamber. The chamber featured an apparatus that applied cyclic stretching (10% elongation) to the cells. Stretching at 10% elongation (15cycles/min, 37°C, 5%CO2) was applied to LF fibroblasts for 12, 24, and 36 h in this study. Following stretch stimulation, total RNA and protein of LF fibroblasts in each chamber were extracted for further experimentation.

### Histological analysis and immunohistological evaluation

Immediately following the surgery, human and rabbit LF samples were preserved in 4% paraformaldehyde for two days. After decalcification for three weeks, the specimens were paraffin-embedded and cut into sections of 4-5µm using (EM FC7, Leica). As directed by the manufacturer, the sections were deparaffinized and hydrated before being stained using EVG staining kits (ZCI BIO, China), H&E staining kits (Yeasen, China), and Masson trichrome staining kits (Yeasen, China). H&E staining was employed to assess the cell density, morphology, and structure of the LF. The elastic fiber to collagen fiber ratio, fibrosis score, and the LF area, thickness, and width were examined through the use of Masson trichrome staining and EVG staining.

Tissue sections were dewaxed and rehydrated before immunohistochemical staining. Then, 10% antigen retrieval solution (AmyJet Scientific Inc, Wuhan, China) was used to retrieve the sections. For 1 hour at room temperature, 10% goat serum (AmyJet Scientific Inc, Wuhan, China) was used to block nonspecific binding after 15 min of blocking with 3% hydrogen peroxide (AmyJet Scientific Inc, Wuhan, China). Subsequently, the sections were incubated with primary antibodies against α-SMA and iNOS (Abcam). Following PBS washing, the sections were treated with secondary antibodies (Abcam) at room temperature. For nuclear counterstaining, Hoechst 33342 was applied at a 1:1000 concentration from Invitrogen. Finally, the images were acquired by Olympus microscope (CX31, Japan) and the results were analyzed by GraphPad Prism 9.5.1 software.

### TEM observation of LF specimens

The ultrastructure alterations of LF were analyzed using a transmission electron microscope (TEM) (Delong, America). LF specimens were gathered, diced into tiny pieces around 1 mm^3^, and submerged in glutaraldehyde solution for three hours. Then, An ultramicrotome (Leica, Germany) was used to slice the specimens into sections of 80nm after dehydrating with propylene oxide and embedding in Epon. The sections were observed and analyzed under the TEM after being stained with 2% uranyl acetate.

### Immunofluorescence staining

In the first step, the LF cells were treated with 4% paraformaldehyde at room temperature for 10 minutes, then permeabilized using Triton X-100 for 15 minutes, and finally blocked with goat serum for 1 hour. Afterward, the cells underwent incubation with antibodies specific to TGF-β, COL1A1, COL3A1, α-SMA, TNF-α, and IL-6 overnight. Finally, the LF cells were exposed to secondary antibodies (Abcam) in dark conditions for 1 hour. The nuclei were stained with DAPI (Beyotime, China) for 10 minutes, and images were taken and analyzed using the fluorescence microscope (Olympus, China). The fluorescence intensity was analyzed by NoviSight software (Shenzhen, China).

### RT- PCR

LF samples from humans and rabbits collected during surgery were promptly frozen and dissolved in TRIzol reagent (Invitrogen, USA) to extract RNA. Next, the extracted RNA served as a template for cDNA synthesis using a Reverse Transcription Kit from Takara, China. Ultimately, the gene expression levels were examined with a Real-Time System (Takara, China). Gene expression quantification was performed by using the 2^-△△CT^ technique. GAPDH was used to perform an internal control. **Table 2** shows the primer sequences for the tested genes.

**Table 2:** RT-PCR primers used in this study.

Primer	Sequence
α-SMA	Forward: 5′ -CATTTGGTCAGAAGACGGTTG-3′ Reverse: 5′ -GACCTGGAGTTCTCACTTTCATC-3′
TGF-β1	Forward: 5′ -TGGACACACAGTACAGCAAGGTCC-3′ Reverse: 5′ -ATCATGTTGGACAACTGCTCCACC-3′
TNF-α	Forward: 5′ -TTATGGCTCAGGGTCCAACTCTGT-3′ Reverse: 5′ -TGGACATTCGAGGCTCCAGTGAAT-3′
IL-6	Forward: 5′ -GCTCTCCTAACAGATAAGCTGGAG-3′ Reverse: 5′ -CCACAGTGAGGAATGTCCACAAAC-3′
GAPDH	Forward: 5′ -AGAAGGTGGTGAAGCAGGCGTC-3′ Reverse: 5′ -AAAGGTGGAGGAGTGGGTGTCG-3′
COL1A1	Forward: 5′ -CAAGACCACCAAGACCTCCCG-3′ Reverse: 5′ -GTCTGGGTTGTTTGTCGTCTGTTTC-3′
COL3A1	Forward: 5′ -CGAGCCTCCCAGAACATCAC-3′ Reverse: 5′ -GAGCAGCCATCCTCCAGAAC-3′

### Western blotting

Total protein was obtained from tissues and cells with the help of a Protein Extraction Sample Kit provided by Sigma. After separating protein samples in 10% SDS-PAGE gels, they were electroblotted onto PVDF membranes (Sigma). Then, the membranes were blocked for 30 minutes at room temperature using a rapid blocking solution (MB-073, Rockland), followed by incubation with primary antibodies against α-SMA (1:1000, 55135-1-AP, Proteintech), TGF-β1 (1:500, ab9758, Abcam), COL3A1 (1:1000, ab184993, Abcam), GAPDH (1:1000, AP0063, Bioworld) at 4°C overnight. Ultimately, the western blotting kit from Bioworld was employed to identify signals of every blotting band following incubation with secondary antibodies (1:1000, SA00001-2, Proteintech).

### ELISA

The concentrations of local TGF-β, iNOS, TNF-α, and IL-6 were analyzed in supernatants gathered from fibroblasts and rabbit LF tissues. According to the manufacturer’s protocol, enzyme-linked immunosorbent assays (ELISA) kits (J20226, Wuhan, China) were used for measurements.

### Statistical analysis

GraphPad Prism software (San Diego, CA, USA). was used to conduct statistical analysis and create graphs. A Student’s t-test was utilized to analyze quantitative data and compare values between two groups. For comparisons among more than two groups, one-way ANOVA tests were applied, with subsequent Tukey’s HSD multiple comparison tests. Pearson correlation analysis was used to examine the relationship between iNOS and LF thickness. At P<0.05, the difference was statistically significant.

## Results

### Anatomy of rabbit spinal structure and modeling methods

Firstly, we conducted anatomical observation and measurement on the lumbar spine of the adult rabbits. It was found that there were 7 vertebral bodies in the rabbit lumbar spine (**Figures 1A, B**), each with one spinous process, two accessory processes, and two transverse processes (**Figures 1A, B**). The body of lumbar spine was small at the proximal end and gradually increased towards at the distal end (**Figure 1B**).

In Locking Plate group, the internal fixation with titanium locking plate (Huasen, Changzhou, China) was positioned on the posterolateral side of L3-4 and L5-6 (5, 6) (**Figures 1C, D**). There was no direct visualization or touching of the LF during fixation procedures so that tissue damage could be minimized. The Steel Wire model in this study improved the previous surgical method by using the steel wire fixation of the spinous process and accessory process (**Figures 1E, F**). This modeling technique offered benefits such as reduced molding costs and increased success rates. It also minimized operation time, decreased exposure depth and technical difficulties, and lessened tissue trauma. (**Table 3**).

**Table 3:** Comparison of data between locking plate and steel wire group.

Index	Locking plate	Steel Wire	P value
Number	12	12	–
Operating time(min)	40 ± 9.5	20 ± 3.4	<0.05
Blood loss(ml)	3.2 ± 1.1	1.5 ± 0.6	<0.05
Incision length(cm)	3.1 ± 0.6	2.0 ± 0.4	<0.05
Spinal cord injury	4 (33.3%)	0	<0.05
Fibrosis score	2.9 ± 0.8	3.0 ± 0.7	>0.05

In BS group, bipedal standing posture was induced in the rabbits by placing them in a rectangular chamber eight hours a day (**Figure 1G**). As described in the **Figure 1H**, we cut the L4/5 posterior lumbar spine at the midpoint of the LF’s upper and lower edges from the axial plane. The LF width, LF area, and LF thickness were measured using the methods illustrated in **Figure 1I**.

### Intervertebral range of motion and mechanical stress loading analysis

**Figure 2:**
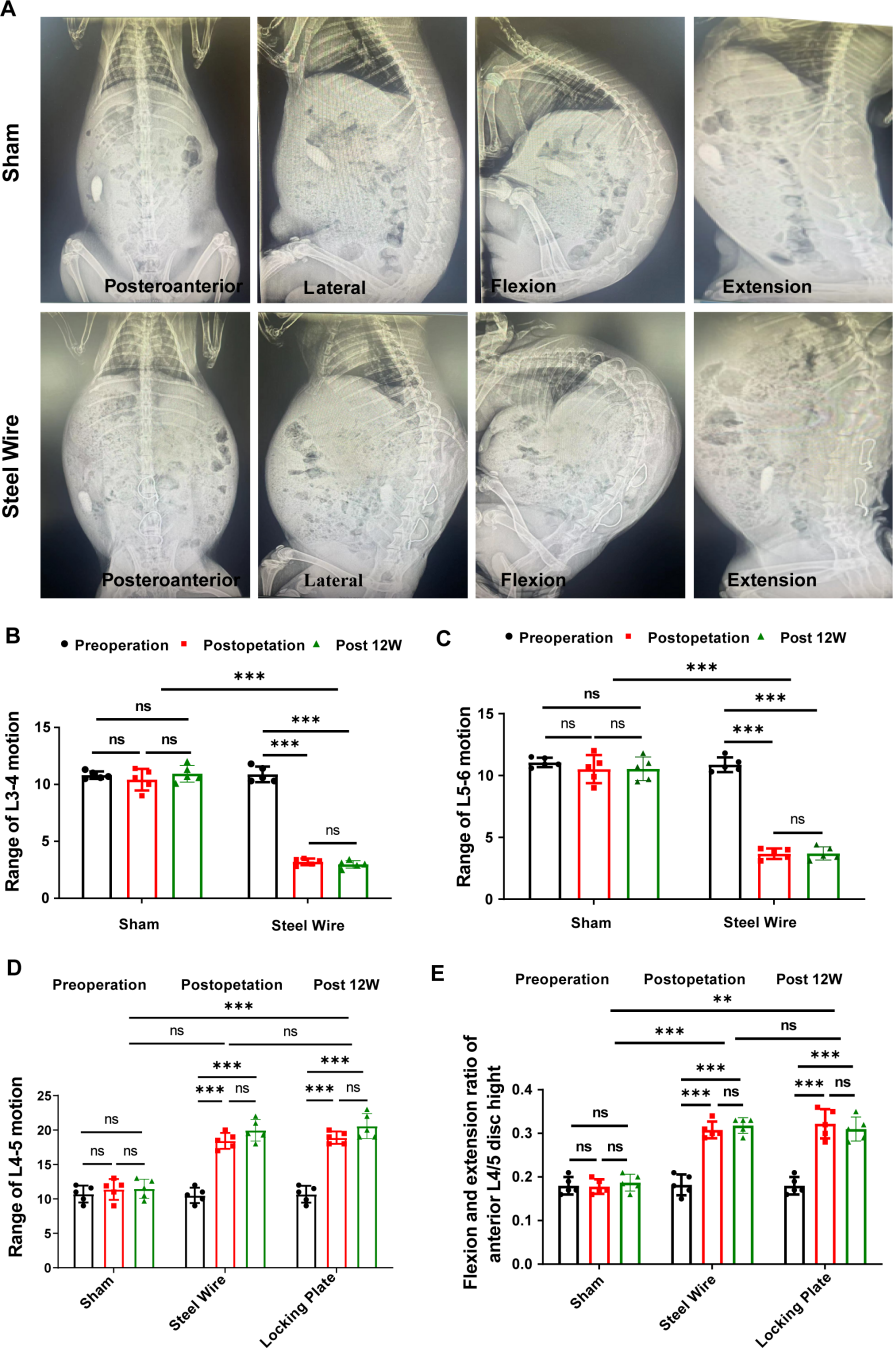
Dynamic evaluation of motion range with X-ray. **(A)** Typical plain X-ray images in posteroanterior, lateral, flexible, and extensional position. **(B–D)** Measurement of movement range of L3-4 **(B)**, L4-5 **(C)** and L5-6 **(D)**. **(E)** Measurement of the anterior L4-5 disc height in the flexion and extension position. ^**^p<0.01,^***^p<0.001, ns, no significance.

To create a rabbit model of LF hypertrophy, we designed this new spinal internal fixation model in which the rabbit LF at the L4/5 level was repeatedly mechanically stressed. As presented in **Figures 2A–D**, measurements and analyses of the motion range for L3/4, L4/5, and L5/6 are conducted in each group. In fusion segment of L3/4 (**Figure 2B**) and L5/6 (**Figure 2C**), nearly all movement range was lost right after surgery and stayed below 5 degrees at 12 weeks post-operation. Accordingly, there was no change in the motion range in L3/4 and L5/6 levels during the study periods. In contrast, the L4-5 range of motion increased significantly right after surgery compared to before surgery. It seemed that an increase was also seen at 12 weeks compared to immediately after surgery, however, no statistical significance was observed (p<0.05; **Figure 2D**). Also, no statistical significance of the L4-5 range motion was observed between the Steel Wire group and Locking Plate group, suggesting the same surgical effect. Moreover, based on the radiological findings, the stress group rabbits (Steel Wire and Locking Plate) exhibited a greater disc space at the anterior vertebral rims of the L4/5 level after surgery than those before surgery, which were maintained at 12 weeks after operation (**Figure 2E**).

### Histological evaluation of human and rabbit LF

**Figure 3:**
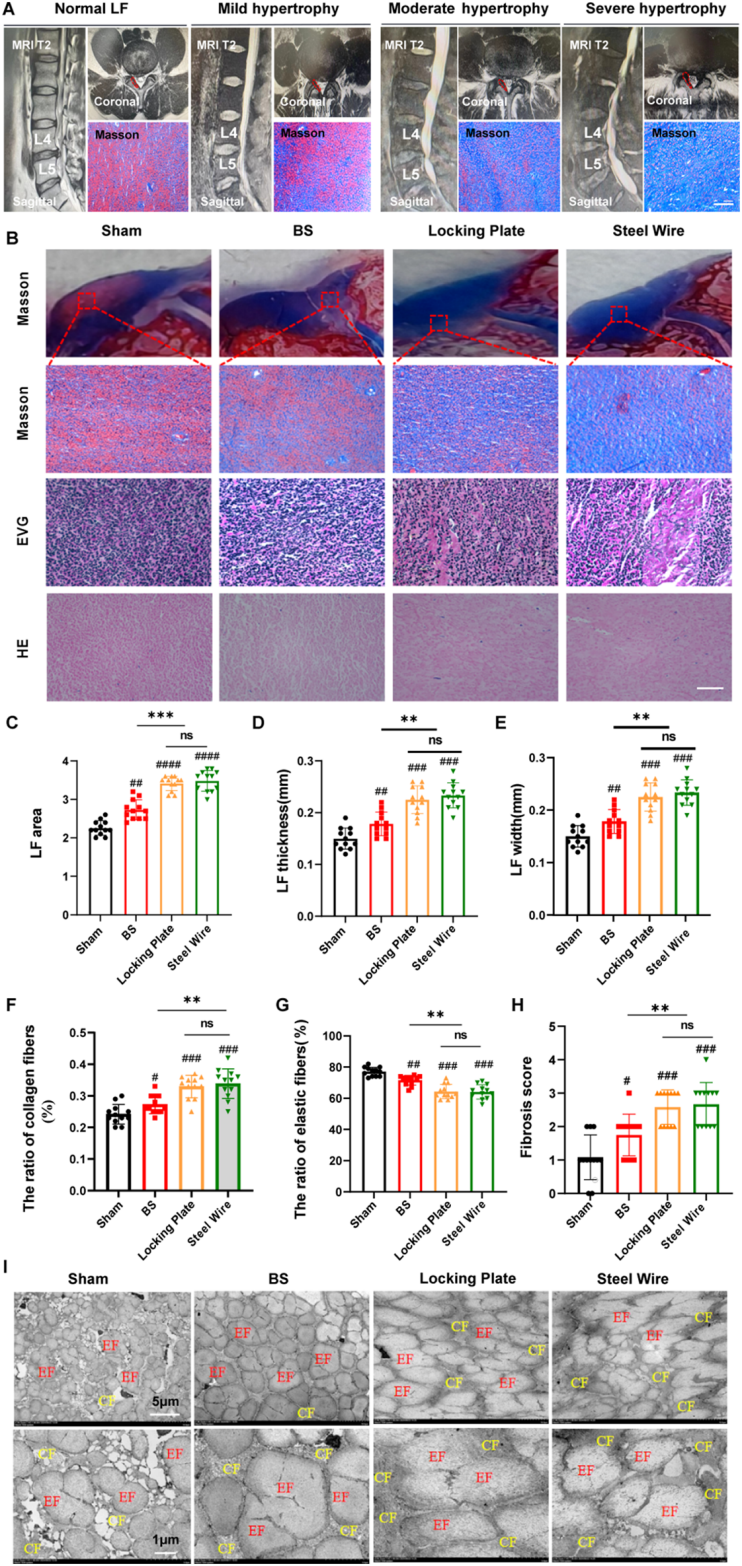
This rabbit model was similar in histology to the moderate LFH observed in humans. **(A)** MRI images and Masson staining display the human LF in states of normal, mild, moderate, and severe hypertrophy, with red lines highlighting the LF outlines. Scale bar, 100μm. **(B)** Images of rabbit LF samples in various groups stained with H&E, EVG, and Masson (n=12). Scale bar, 50μm. **(C)** Measurement and analysis of the LF area with Masson staining (n=12). **(D)** Analyzing the LF thickness among the four distinct groups (n=12). **(E)** Assessment of LF width in the four groups (n=12). **(F, G)** Evaluation of the area proportions of collagen and elastic fibers (n=12). **(H)** Comparison of the fibrosis scores in the four groups (n=12). **(I)** Rabbit LF ultrastructure was examined with transmission electron microscopy (n=4). Results are shown as mean ± standard deviation; ^**^P< 0.01;^***^P<0.001; ^#^P<0.05; ^##^P<0.01; ^###^P<0.001. BS, bipedal standing; LF, ligamentum flavum; EF, elastic fiber; CF, collagen fiber; ns, no significance.

To assess the feasibility of using this surgical model as an experimental model for exploring the pathological mechanism of LF hypertrophy, we conducted histological analyses of the rabbit lumbar spine. Firstly, we assessed the extent of LFH in rabbits and compared it to human samples. To measure LF thickness in humans, coronal T2-weighted MRI images at the L4/5 facet joints were employed (**Figure 3A**). For patients with LSCS, the thickness of LF is often over 4 mm (14, 15). In **Figure 3A**, representative MRI scans indicated that LF thicker than 4mm typically compressed the dural sac and nerve roots. In typical LF patients without hypertrophy, the dural sac was not compressed and generally measured under 4 mm in thickness. As the LF thickened, it caused the spinal canal to narrow and increased the severity of LF fibrosis.

Histologically, elastic fibers in the non-hypertrophied LF of humans were abundant and systematically organized, whereas severe hypertrophy of LF showed uneven, fragmented, irregularly arranged, and partially absent elastic fibers in Masson staining (**Figure 3A**). Moreover, the ratio of elastin to collagen significantly dropped in hypertrophied LF compared to normal LF (**Figure 3A**). In our rabbit model, the control group showed dense and aligned elastic fibers, while the Steel Wire group exhibited moderate degeneration and a reduced elastin-to-collagen ratio (**Figures 3B, F, G**). Quantitative analysis showed that the LF area (**Figure 3C**), thickness (**Figure 3D**), and width (**Figure 3E**), and fibrosis score (**Figure 3H**) in the Steel Wire group was notably higher compared to the control group at the same level. Furthermore, TEM analysis of LF ultrastructure revealed that the control group had a high abundance of elastic fibers, with most exhibiting a long shuttle or irregular shapes observed in the coronal plane. Nevertheless, the elastic fibers in the Steel Wire group was decreased and were mostly characterized by short shuttle and elliptical shapes (**Figure 3I**). The findings above indicate that the rabbit model subjected to mechanical stress in the Steel Wire group shows fibrotic alterations and is histologically similar to moderate LFH in humans.

To further evaluate the effect of our modeling, we compared our surgical model with the existing ones. It was found that the LF in bipedal standing rabbits was found to be histologically similar to mild hypertrophy in human LF. Moreover, the histological changes in LF from the surgical model groups (Locking Plate and Steel Wire) were comparable, matching the moderately hypertrophied LF found in humans (**Figures 3A, B**).

### Changes in cell type and activity in the hypertrophied LF

**Figure 4:**
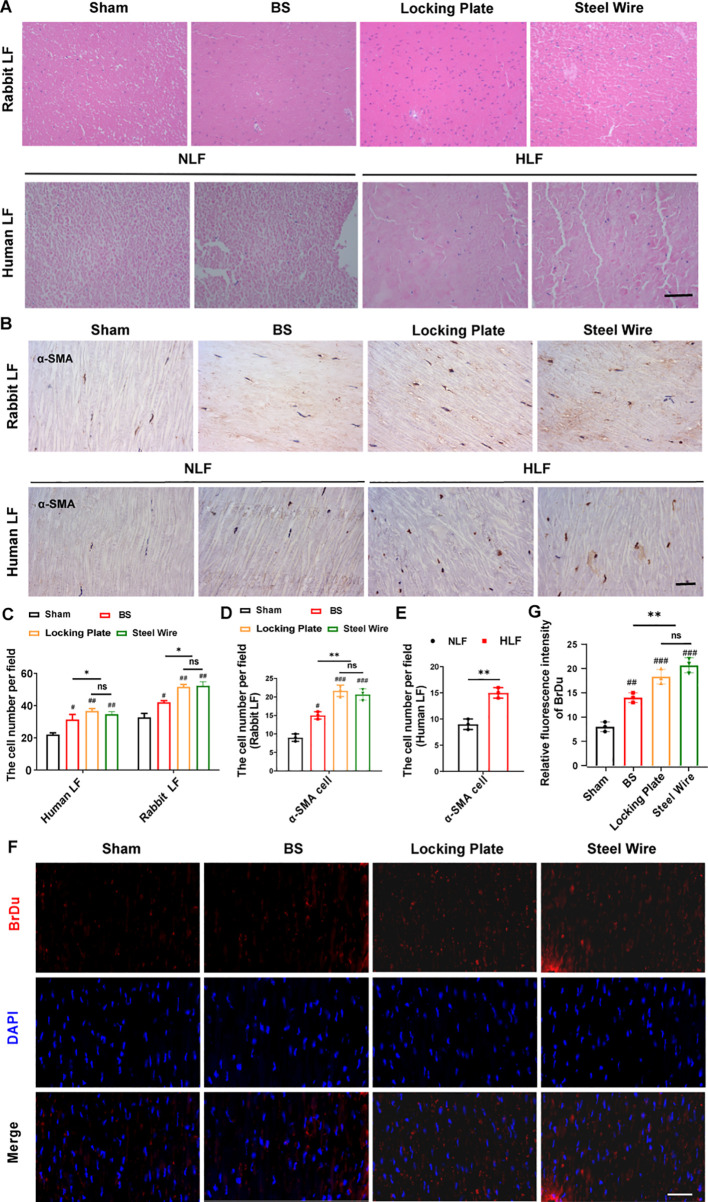
Variations in cell type and activity within the hypertrophied LF. **(A)** The representative image of H&E staining reveals alterations in cell density in rabbit and human LF samples across different groups. Scale bar, 100μm. **(B)** Immunohistochemical analysis of α-SMA-positive cells in LF samples from rabbits and humans. Scale bar, 25μm. **(C)** Quantitative evaluation of LF cell density across each field (n=5). **(D)** Quantitative assessments of α-SMA-positive cells in each high power field of human LF (n=5). **(E, F)** The proliferative activity of cells in rabbit LF identified by BrDu. Scale bar, 25μm. ^*^P<0.05, ^**^P<0.01; ^#^P<0.05, ^##^P<0.01, ^###^P<0.001. BS, bipedal standing; NLF, normal ligamentum flavum; HLF, hypertrophied ligamentum flavum; ns, no significance.

As is known, the increase of the cell density means that the number of cells increases, and the interactions between cells are also enhanced, thereby promoting communication and signal transmission between cells. The rise in cell density might also be linked to biological processes like cell proliferation and differentiation, which can affect tissue development and repair. At the same time, it may also involve the interaction, mutual influence, and mutual regulation of different cell types (16, 17). Thus, to investigate more deeply the pathological mechanism behind LFH, we next examined the changes in cellular distribution using samples taken from humans and rabbits. Rabbit non-hypertrophied LF contained few cells, while rabbit hypertrophied LF showed a significant increase in cell numbers, which was in according with the observation in human LF (**Figures 4A, C**). Furthermore, there was a notable increase in BrdU-positive proliferating cells in both the Steel Wire and locking Plate groups compared to the control group (**Figures 4F, G**). In summary, LFH was significantly related to changes in both cell density and cellular activity.

During fibrosis, fibroblast-myofibroblast transition affects the production of fibrosis-related factors and ECM, which drives diverse tissue and organ fibrosis responses (18, 19). The expression of α-SMA is a characteristic of activated myofibroblasts (20). Also, in our rabbit model, the stress group (Locking Plate and Steel Wire) had a significantly greater number of ɑ-SMA-positive LF cells compared to the control group (**Figure 4B, D, E**), indicating the important role of myofibroblasts in LF fibrosis. This cellular pathology of hypertrophic LF in the our stress group is comparable to that in humans.

### Mechanical stress promotes LF fibrosis by inducing local inflammation

**Figure 5:**
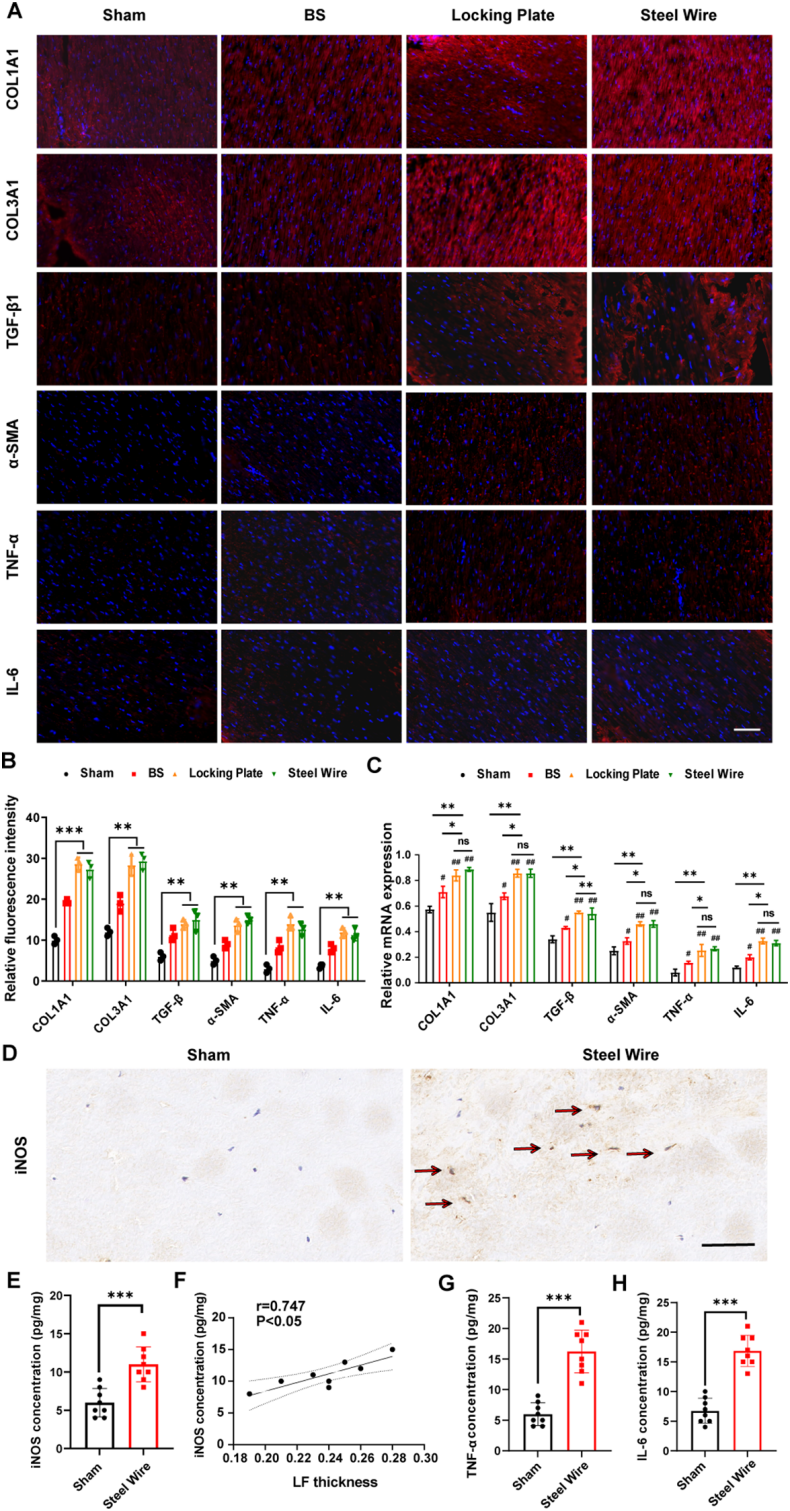
Mechanical stress promotes LF fibrosis by triggering local inflammation. **(A)** Red immunofluorescence staining on LF sections displayed COL1A1, COL3A1, TGF-β1, α-SMA, TNF-α, and IL-6. Scale bar, 50μm. **(B)** Quantitative evaluation of the fluorescence density of COL1A1, COL3A1, TGF-β1, α-SMA, TNF-α, and IL-6. **(C)** The mRNA levels of inflammatory cytokines and fibrosis-related factors analyzed through RT-PCR. **(D)** Representative images showing M1 markers (iNOS) detected using immunohistochemical staining. Scale bar, 50μm. **(E)** Expression levels of M1 makers (iNOS) in rabbit LF tissues as detected by ELISA. **(F)** Analysis of the correlation between iNOS protein levels and LF thickness. **(G, H)** The levels of TNF-a and IL-6 in rabbit LF tissues identified through ELISA. ^*^P < 0.01, ^**^P<0.05, ^***^P< 0.0001; ^#^P<0.05, ^##^P<0.01. BS, bipedal standing; ns, no significance.

To explore the molecular mechanism of mechanical stress LF fibrosis, we measured the levels of collagen, growth factors, and inflammatory cytokines in our rabbit model. Immunofluorescence staining exhibited that the Locking Plate group and Steel Wire group accumulated more collagen (COL1A1 and COL3A1) compared to the control group (**Figures 5A–C**). This findings aligned with histological studies, further illustrating fibrotic changes in rabbit LF.

There is increasing proof that inflammation is a key factor in the development of fibrosis (21, 22). Consistent with these findings, ongoing chronic inflammation also leads to progressive LF fibrosis (23, 24). As such, an obvious elevation of inflammatory cytokines, including TNF-α and IL-6, measured by immunofluorescence staining and RT-PCR were also observed in the stress group (Locking Plate group and Steel Wire group, **Figures 5A–C**), which indicated the association of mechanical stress with local inflammation in LFH.

Recently, macrophages have been found to play a role in the advancement of fibrosis in various organs (25–27). After pathological tissue injury, macrophages adjust their morphological and functional traits through phenotypic and functional plasticity (28, 29). A macrophage can be classified into a variety of phenotypes based on its heterogeneity. M1 macrophages are important for inducing an inflammatory response by releasing pro-inflammatory cytokines, including TNF-α, IL-6, and IL-1β (28, 29). To explore the correlations between macrophage infiltration and LF fibrosis, we assessed LF thickness based on Masson staining and identified macrophage infiltration with immunohistochemical staining and ELISA. From the results, we demonstrated that the proportion of M1 detected by immunohistochemical staining was obviously increased in hypertrophic LF samples (Steel Wire group) compared to the control LF samples (**Figure 5D**). Consistently, the findings of ELISA indicated that the LF in Steel Wire group exhibited an increase in the protein expression of M1 makers (iNOS) (**Figure 5E**). Furthermore, positive correlations were found between the protein levels of M1 makers (iNOS) and LF thickness (**Figure 5F**). Simultaneously, there was an increase in the expression of inflammation-related genes TNF-α and IL-6 in Steel Wire group compared to the control group (**Figures 5G, H**). In conclusion, the above results suggested that M1 macrophage infiltration were associated with local inflammation and thereby played a crucial role in the progression of LFH.

### Mechanical stress induces LF fibrosis through promoting the fibroblast-to -myofibroblast transition

**Figure 6:**
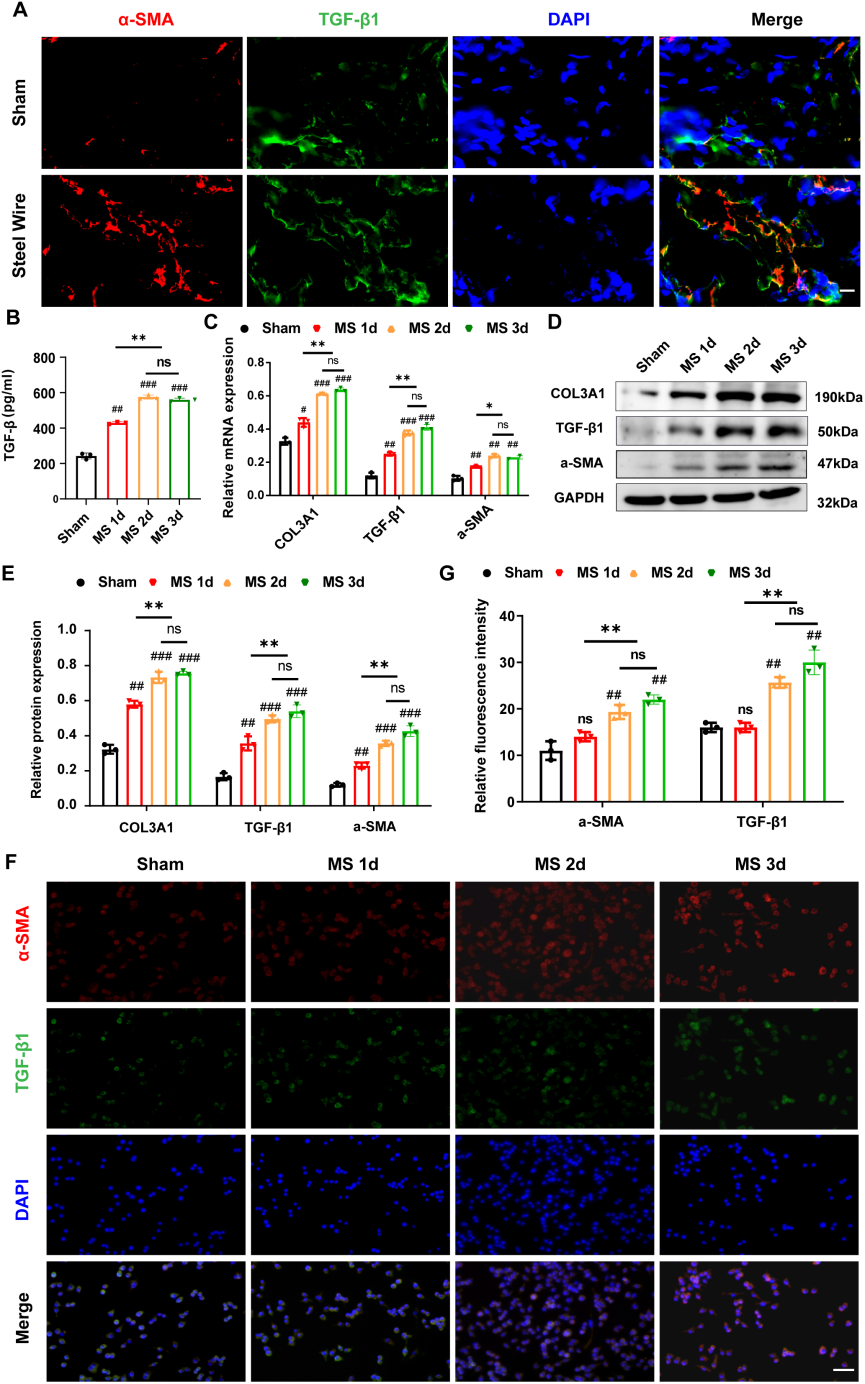
Mechanical stress induce LF fibrosis through promoting the fibroblast-to-myofibroblast transition. **(A)** The distribution of α-SMA (red) and TGF-β1 (green) in LF from the Steel Wire and control groups shown by double-immunofluorescence staining. Scale bar: 10μm. **(B)** TGF-β1 levels in the supernatants of different modes detected by ELISA. **(C)** Expression of fibrosis-associated proteins (COL3A1, TGF-β1, and α-SMA) after mechanical-stretch stimulation in different time-points detected using RT-PCR. **(D, E)** Analysis of fibrosis-related protein expression using Western blot after mechanical stretch at several time points. **(F, G)** Double-immunofluorescence staining of fibroblast after stimulation by mechanical stretch reveals the TGF-β1 (green) and α-SMA (red). Scale bar: 25μm. Mean ± SD results are presented; ^*^P <0.05, ^**^P <0.01, ^***^P <0.001, ^****^P <0.0001; ^##^P < 0.01, ^###^P < 0.001. MS, mechanical stretch; ns, no significance.

As demonstrated above, hypertrophied LF exhibited markedly elevated a-SMA expression levels detected by immunohistochemistry staining (**Figure 4B**), consistent with previous literature reports (5–7). Additionally, studies have shown that increased levels of TGF-β are significantly observed in hypertrophied LF tissues from LSCS patients. Substantial evidence suggests that TGF-β1 enhances the production of ECM proteins, especially collagen, in LF cells, highlighting its significant role in LF fibrosis (30, 31). Similarly, in our Steel Wire group, TGF-β1 levels identified through immunofluorescence staining (**Figure 5A**) were notably higher than those in the control group. The activation of myofibroblasts linked to TGF-β signaling has been extensively documented. Myofibroblasts possess enhanced ability to synthesize ECM and participate in tissue remodeling (32, 33). Importantly, our immunofluorescence analysis of LF tissues revealed that a relative higher number of LF cells with fibrosis features (TGF-β1+α-SMA), known as myofibroblasts, were present in the hypertrophic LF in Steel Wire group (**Figure 6A**). This finding shows that fibroblasts with high plasticity can transform into myofibroblasts by the exist of TGF-β1, suggesting mechanical stress/TGF-β1/α-SMA axis may be crucial in mediating the progression of LF fibrosis.

Furthermore, in order to explore the mechanism of mechanical stress/TGF-β1/α-SMA axis involved in the development of LFH, we *in vitro* created an experimental apparatus that allows for the application of repeated cyclic mechanical stretch to LF fibroblasts sourced from rabbit LF. As expected, the TGF-β level in the supernatant elevated under the stimulation of cyclic mechanical stretch in the present study. When mechanical stress was prolonged, LF fibroblasts produced more TGF-β1 in their supernatants detected by ELISA (**Figure 6B**). Additionally, Stretch stimulation significantly increased the expression of TGF-β1 and α-SMA in LF fibroblasts, and these increases appeared to be time-dependent (**Figures 6C–E**). With prolonged duration of mechanical stretch, most fibroblast cells transform into the myofibroblast phenotype (**Figures 6C–E**), which was also confirmed by immunofluorescence analysis (**Figures 6F, G**), indicating that mechanical stretch plays a crucial role in the transition from fibroblast to myofibroblast by promoting TGF-β1 secretion. Consistently, the COL3A1 protein levels in LF cells, as shown in **Figures 6C–E**, increased over time when subjected to mechanical stretching, as detected by mRNA and western blotting. Collectively, our conclusion is that persistent mechanical stress boosts the release of TGF-β1 in LF fibroblasts and some inflammatory cells, which induces the transition of fibroblast-to-myofibroblast and finally results in collagen accumulation and LF fibrosis.

## Discussion

Degeneration of the disk and facet joints typically leads to segmental instability in individuals with LSCS. This increasing instability plays a crucial role in LFH pathogenesis (34, 35). However, the specific mechanism of mechanical stress-induced fibrosis of the LF is not very clear. By subjecting the rabbit LF to continuous mechanical stress, we also determined that mechanical stress independently causes LFH. Moreover, in our rabbit stress model, disordered organization structure, collagen accumulation, changes in cellular components, and the unusual expression of factors linked to fibrosis were observed, which was histologically identical to moderate LFH in humans. Furthermore, it was found that sustainable mechanical stress promoted LF fibrosis by inducing local inflammation and fibroblast-to- myofibroblast transition. Collectively, our results indicate that prolonged mechanical stress causes recurrent micro-injuries in rabbit LF tissues, which stimulates local inflammation and TGF-β1 secretion, subsequently induces myofibroblast transition, ultimately leading to LFH.

Currently, the construction of a recognized model of lumbar LF hypertrophy is still in its exploratory stages. Various studies have conducted many experimental studies on animal models, but the current models have varying degrees of defects. Non-surgical modeling techniques of LF hypertrophy involve a mouse model employing an innovative loading device to impose constant mechanical stress on the lumbar LF (36) and the bipedal standing mice (37). Nonetheless, these two models demand specialized equipment and involve a lengthy molding process, making them challenging to manage. LFH might also be triggered by damaging the spinous process along with ligaments, paraspinal muscles, and facet joints, thereby focusing more stress on a specific lumbar LF (12). The drawbacks involve considerable trauma and increased bleeding. Simultaneously, the LF displays only minor hypertrophic pathology. Another surgical animal model of intervertebral mechanical stress concentration using posterolateral fusion with instrumentation is also proposed (11). Similarly, significant tissue damage, high costs, and greater technical complexity characterize this model type, thereby limiting its application to some degree. Consequently, developing a better pathological model will be crucial for understanding the pathological mechanisms and for preventing and treating LFH. In this study, we effectively created a rabbit model of LFH using a new internal fixation technique. Briefly, the rabbit was performed posterior fusion with steel wire to fix spinous and accessory processes. The pathological traits of the model were quite similar to those of moderate human LFH. Additionally, this technique of rabbit model was not difficult and easy to operate. In brief, this rabbit model shortened the operation time, reduced exposure depth and modeling costs, and decreased the tissue trauma as compared with the traditional locking plate fixation, which was an ideal animal model for studying LFH.

After tissues are injured by pathology, macrophages play a vital role in modulating morphological and functional characteristics through phenotypic and functional plasticity (28, 29). Inflammation and tissue damage repair have been the major focus of previous studies involving macrophages (38). Currently, more and more studies have shown that macrophages infiltration has been implicated in various forms of fibrosis (25–27). The regulation of macrophages has been found to be involved in inflammation and fibrosis formation (39, 40). Here, it was demonstrated that infiltration of iNOS-positive macrophages and high levels of inflammatory cytokines, including TNF-α and IL-6, occurred in the LF tissues from the Steel Wire group. Hence, we deduce that ongoing mechanical stress at the LF might lead to micro-injury in daily routines. As a consequence, inflammation occurs, which involves inflammatory macrophages and other cells that secrete some factors to coordinate the healing process. When the micro-injury continues, it might result in the development of a thick fibrotic mass.

Fibroblasts generate a variety of structural and adhesive proteins for the extracellular matrix, marked by the presence of collagen I, N-cadherin, and vimentin (41). In fibrosis, the transition from fibroblasts to myofibroblasts, which generate ECM and release factors related to fibrosis, is a crucial event that drives the fibrotic response (19, 20). It has been widely recognized that TGF-β1 signaling is involved in myofibroblast transition (42, 43). Interestingly, it is demonstrated that myofibroblasts and fibroblasts also co-exists in hypertrophied LF tissues (44). Furthermore, the presence of exogenous TGF-β1 accelerates the differentiation of fibroblasts into myofibroblasts (45). In this study, a higher number of LF cells with fibrosis features (TGF-β1+α-SMA) were evidenced in the hypertrophic LF in our Steel Wire group. Also, *in vitro*, the expression of α-SMA protein in LF cells significantly increased with prolonged mechanical stress. Therefore, it is speculated that mechanical stress could enhance the secretion of TGF-β1 in LF fibroblasts, subsequently leading to the myofibroblast transition. As expected, it was found that stretch stimulation *in vitro* significantly increased both the expression of TGF-β1 and α-SMA in LF fibroblasts. Consequently, it may be inferred that mechanical stress-induced transdifferentiation of fibroblasts into myofibroblasts is also a significant step in LF fibrosis. Hence, devising a strategy to identify novel drugs aimed at TGF-β1 intervention in this innovative model might be a beneficial research avenue for the reversibility of LF fibrosis. Due to the widespread reports of drug resistance in fibrotic tissue, further research is necessary to validate this potential application.

There are several limitations to this study. Firstly, each group only has 8 rabbits, indicating that larger samples are needed to validate this findings. Secondly, the progression of fibrosis is influenced by multiple mechanisms, and it is still uncertain whether additional factors are involved in LF hypertrophy. Thirdly, our rabbit model is histologically identical to moderate LFH of human. Whether extending the modeling time could achieve severe LFH is an area of investigation that needs to be further explored.

**Figure 7:**
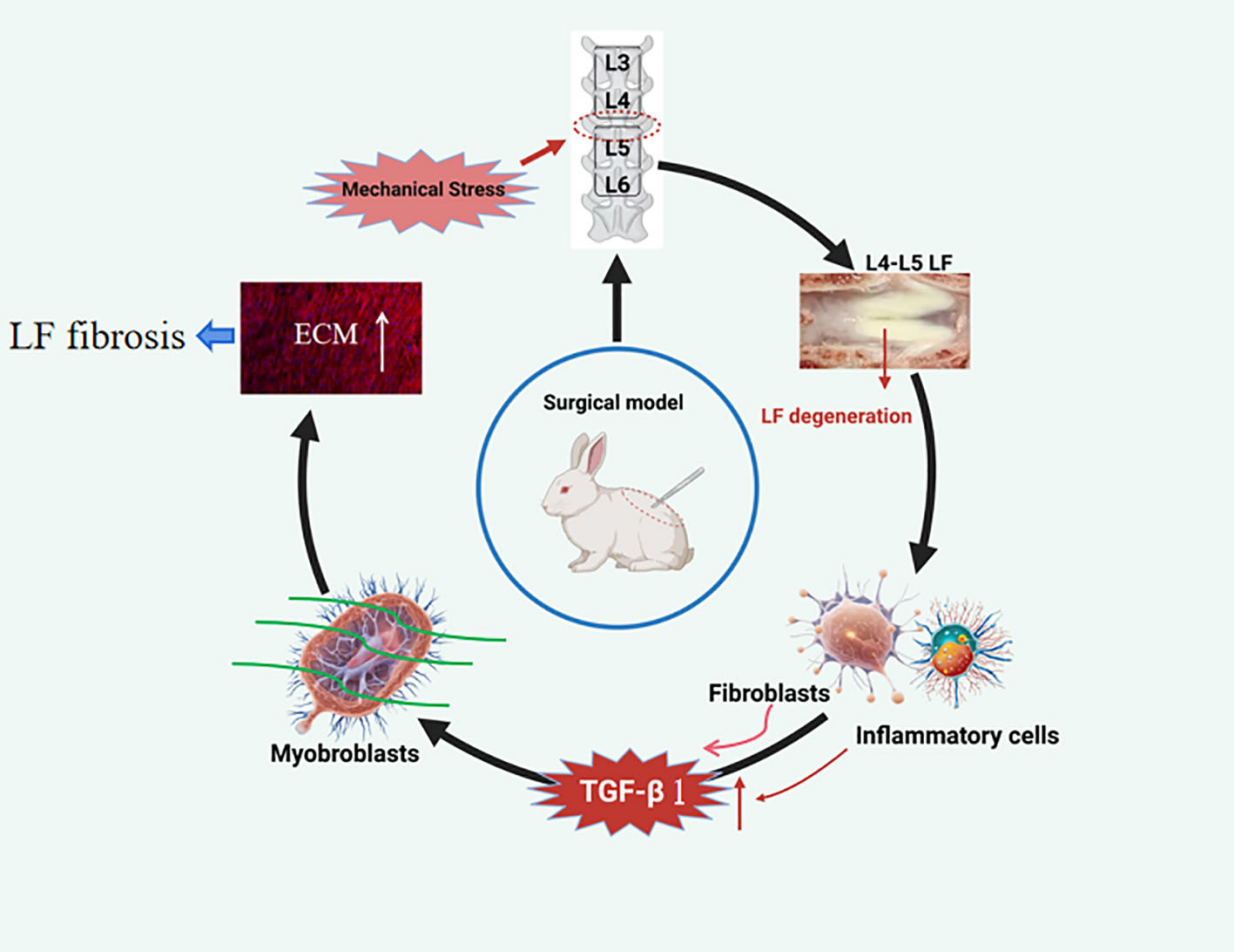
Proposed model depicting the mechanical stress-induced mechanism of LFH. Continuous mechanical stress causes micro-injuries and local inflammation in the LF, resulting in increased TGF-β1 secretion by LF fibroblasts and certain inflammatory cells. Subsequently, the increased TGF-β1 induced the transition of fibroblasts into myofibroblasts, finally resulting in collagen accumulation and LFH.

To conclude, we present an innovative rabbit model for studying the pathological mechanism of LFH. LFH with increase in collagen, decrease in elastic fibers, and changes in cellular components is observed in this steel wire model, which is histologically similar to moderate LFH of human and superior to traditional locking plate fixation surgery in lowering modeling expenses, minimizing trauma, and reducing technical challenges, suggesting that this animal model is ideal for studying LF hypertrophy. Mechanistically, prolonged mechanical stress causes micro-damage and local inflammation in LF tissues, which then triggers the release of TGF-β1 in LF fibroblasts and certain inflammatory cells, and causes fibroblast-to-myofibroblast transition, ultimately resulting in LF fibrosis and hypertrophy (**Figure 7**).

## Data Availability

The raw data supporting the conclusions of this article will be made available by the authors, without undue reservation.
